# A Transgenic Tri-Modality Reporter Mouse

**DOI:** 10.1371/journal.pone.0073580

**Published:** 2013-08-09

**Authors:** Xinrui Yan, Pritha Ray, Ramasamy Paulmurugan, Ricky Tong, Yongquan Gong, Ataya Sathirachinda, Joseph C. Wu, Sanjiv S. Gambhir

**Affiliations:** 1 Departments of Radiology, MIPS and Bio-X, Stanford University, Stanford, California, United States of America; 2 ACTREC, Tata Memorial Centre, Kharghar, Navi Mumbai, India; 3 Stanford Cardiovascular Institute, Stanford University, Stanford, California, United States of America; 4 Departments of Bioengineering and Materials Science & Engineering, Stanford University, Stanford, California, United States of America; University of Cincinnati, United States of America

## Abstract

Transgenic mouse with a stably integrated reporter gene(s) can be a valuable resource for obtaining uniformly labeled stem cells, tissues, and organs for various applications. We have generated a transgenic mouse model that ubiquitously expresses a tri-fusion reporter gene (fluc2-tdTomato-ttk) driven by a constitutive chicken β-actin promoter. This “Tri-Modality Reporter Mouse” system allows one to isolate most cells from this donor mouse and image them for bioluminescent (fluc2), fluorescent (tdTomato), and positron emission tomography (PET) (ttk) modalities. Transgenic colonies with different levels of tri-fusion reporter gene expression showed a linear correlation between all three-reporter proteins (R^2^=0.89 for TdTomato vs Fluc, R^2^=0.94 for Fluc vs TTK, R^2^=0.89 for TdTomato vs TTK) *in vitro* from tissue lysates and *in vivo* by optical and PET imaging. Mesenchymal stem cells (MSCs) isolated from this transgenics showed high level of reporter gene expression, which linearly correlated with the cell numbers (R^2^=0.99 for bioluminescence imaging (BLI)). Both BLI (R^2^=0.93) and micro-PET (R^2^=0.94) imaging of the subcutaneous implants of Tri-Modality Reporter Mouse derived MSCs in nude mice showed linear correlation with the cell numbers and across different imaging modalities (R^2^=0.97). Serial imaging of MSCs transplanted to mice with acute myocardial infarction (MI) by intramyocardial injection exhibited significantly higher signals in MI heart at days 2, 3, 4, and 7 (p<0.01). MSCs transplanted to the ischemic hindlimb of nude mice showed significantly higher BLI and PET signals in the first 2 weeks that dropped by 4^th^ week due to poor cell survival. However, laser Doppler perfusion imaging revealed that blood circulation in the ischemic limb was significantly improved in the MSCs transplantation group compared with the control group. In summary, this mouse can be used as a source of donor cells and organs in various research areas such as stem cell research, tissue engineering research, and organ transplantation.

## Introduction

Over the last decade, regenerative medicine with the use of stem cells has appeared as a potential therapeutic alternative for diseases in different organs/systems, such as endocrine, musculoskeletal, and the cardiovascular system [1–4]. However, we still lack a comprehensive knowledge of regenerative mechanisms, especially, in the early phases of treatment. Recent advances in molecular biology and imaging have allowed for the successful non-invasive monitoring of transplanted stem cells in the living subject by labeling transplanted cells. Direct labeling strategies appear to be a good imaging method for detection of cells shortly after transplantation, providing a good signal-to-noise ratio, but less suited for long-term monitoring of stem cell viability [5]. The development of reporter gene strategies made it possible to accurately study the biology of stem cells based on the physiologic activity of transplanted cells [6–10]. In particular the ability of transducing reporter transgenes in non-dividing cells, especially lentivirus was successfully used for reporter gene delivery to the stem cells in many previous studies [11]. However, gene transfer efficiency remains an obstacle, especially for primary cells, tissues and intact organs. Moreover, stable expression of reporter genes in particular cell types over time can be variable. Thus, current methods of introducing reporter genes are not satisfactory for transplantation studies.

More recently, investigators have developed transgenic mouse with stably integrated reporter gene cassettes controlled by promoter which are largely constitutive, thus meeting the above criteria and providing a source of uniformly labeled biological materials for transplantation studies. Bioluminescence imaging (BLI) was previously used to noninvasively visualize engraftment, survival, and rejection of transplanted tissues from a transgenic donor mouse that constitutively expresses luciferase [12]. Soon after, this group has developed a donor transgenic mouse line with a biscistronic gene consisting of two reporter genes, firefly luciferase and enhanced green fluorescence protein (eGFP). By using this *in vivo* imaging approach the investigator has studied the hematopoietic cell reconstitution in the spleen and bone marrow in the mice after irradiation by transplantation [13]. The BLI approach offers the advantage of relatively low cost instrumentation, high throughput, and low background signal. However, the optical approaches suffer from significant photon attenuation, lack of tomographic detail, and lack of generalizability to human studies. Positron emission tomography (PET) has the advantage of being applicable to all living subjects with high sensitivity and good spatial resolution, as well as is tomographic in nature. However, compared to the other modalities, it is somewhat limited by higher cost and the need for a cyclotron for production of isotopes for most PET tracers. Fluorescent imaging allows for high resolution optical imaging using confocal or multiphoton microscopy. It can also be incorporated in histology analysis. Thus each of these modalities has unique applications, advantages, and limitations that can be complementary to the other modalities. A specific strategy for combining the different modalities, including a cell-based technique with an animal imaging technique, is to build a unified fusion gene composed of different reporter genes whose expression can be imaged with different imaging modalities in both individual cells and in living subjects. Our lab has recently constructed and validated the use of several multimodality fusion reporter genes, which combine fluorescence, bioluminescence, and PET imaging [14–16]. Among all of our tri-fusion vectors, the fluc2-tdTomato-ttk which uses an improved bioluminescent (fluc2), red fluorescent (tdTomato) and a truncated herpes simplex virus type 1 sr39 thymidine kinase (ttk) reporter genes, was the most sensitive vector, and so this vector was used to create a transgenic mouse described in this study [17].

Mesenchymal stem cells (MSCs) were first identified in the bone marrow by Fridenshtein [18], and later its presence was reported in various other organs and tissues. MSCs have demonstrated significant potential for clinical use due to their convenient isolation from same patient, lack of significant immunogenicity, and their potential to differentiate into tissue-specific cell types, and promotes vascularization [19,20]. However, the survival rate of exogenously administered cells within the injured tissue remains low, and the mechanisms of stem cell therapy are still unclear. Hence understanding the MSCs survival and proliferation after transplatation is essential for successful development of MSCs based therapies. Several imaging modalities such as, BLI, magnetic resonance imaging (MRI), PET, and fluorescence imaging have been successfully used for the investigation of MSCs fates and functions. However each of these imaging modalities has its own advantages and disadvantages [21–23]. In this study, we inserted the fluc2-tdTomato-ttk fusion gene into pCAGGS (containing the chicken beta-actin promoter and cytomegalovirus enhancer, beta-actin intron and bovine globin poly-adenylation signal) [24] and produced a unique “universal reporter” transgenic mouse model expressing a multimodality fusion reporter gene from a constitutive promoter. This transgenic mouse model was further evaluated for maintaining the constant expression level of reporter gene over time, uniformity in expression across different tissues, and cell transplantation studies particularly for MSC mediated therapeutic effects on myocardial infarction and hindlimb ischemia models.

## Materials and Methods

### Generation and Screening of Tri-fusion Transgenic Mouse

The complete 4.4 kb fragment of fusion reporter (fluc2-tdTomato-ttk) gene was released from pcDNA- CMV-fluc2-tdt-ttk vector by *NheI* and *NotI* double digestion, and then ligated to pCAGGS vector digested with respective enzymes. The pCAGGS vector was earlier modified by addition of suitable restriction enzyme sites through a small nucleotide linker. Selected clones were verified by restriction digestion, and further by sequencing [17].

The tri-fusion transgenic mouse line was made using FVB background. Tri-fusion transgenic was made by pronuclear injection of CAGGS-fluc2-tdTomato-ttk reporter gene fragment from pcAGGS-fluc2-tdTomato-ttk vector with the help of the transgenic animal facility at Stanford University. Injected eggs were re-implanted by oviduct transfer into outbred pseudopregnant females generated by mating to vasectomized males. After the microinjections and oviduct transfers, mice were housed in the facility until pups were weaned at 21 days after birth. Pups were screened by *in vivo* BLI as described in the following paragraph. The positive mice were then screened for the fluorescence signal using *in vivo* fluorescence imaging, and finally for ttk expression by ^18|^F-FHBG ((9-[4-[(18) F]-fluoro-3-(hydroxymethyl) butyl] guanine) micro-PET imaging. We bred all the positive transgenic mice for two generations, and then bred only strongly positive mice with wild type littermate over several generations.

### Characterization of Transgenic Mouse by Bioluminescence, Fluorescence, and MicroPET Imaging

For BLI, transgenic mice were injected intraperitoneally with D-Luciferin (150 mg/kg body weight, Biosynth AG, Itasca, IL). Ten min later, the mice were anesthetized with isoflurane (2% in 1 L/min oxygen) and bioluminescence images were acquired for 1 second using the IVIS 200® system (Caliper Life Sciences, Alameda, CA). Data was analyzed using Living Image 3.2 Software (Caliper Life Sciences).

For fluorescence imaging, **t**he spectral fluorescence images were obtained using the Maestro In-Vivo Imaging System (Cry, Inc., Woburn, MA). Multispectral imaging (MSI) data sets (cubes) were acquired with images spaced every 10 nm spectral interval in the 550 to 700 nm spectral range (excitation filter, 503–550 nm; emission filter, 550–800 nm). The spectral fluorescence images consisting of autofluorescence spectra and tdTomato spectra were obtained and then unmixed, based on their spectral patterns using commercial software (Maestro software, Cry).

Small animal PET imaging studies were performed using a microPET-R4 (concorde Microsystems) scanner. Transgenic mice were anesthetized, and tail vein injected with tracer ^18^F-FHBG (7.4 MBq in 200 µL saline). MicroPET scanning was performed one hour after injection. The microPET images were reconstructed with the ordered-subsets expectation maximization algorithm and analyzed using a Medical Imaging Data Examiner. The radiotracer uptake levels were determined and expressed as percentage injection dose per gram tissues (% ID/g).

### *In vitro* HSV1-Sr39tk Enzyme Activity Assay, Luciferase Assay, Fluorescence Assay and β-Galactosidase Assay

Cells transiently transfected with pcAGGS-fluc2-tdTomato-ttk vector along with β-galactosidase vector or organs from transgenic mice were harvested and homogenized in either luciferase or TK lysis buffer as described previously [25]. Cell/tissue lysates were then assessed for protein concentration by Bio-Rad protein assay reagent (Bio-Rad Laboratories, Hercules, CA) and reading absorbance at 595 nm on a Synergy 4 Microplate reader (Biotek, Winooski, VT). The HSV1-TK reaction buffer was added, and then the reaction mixture was doted to DE81 filters (Whatman). The total amount of phosphorylated penciclovir attached to the filter was determined by scintillation counter and normalized by protein concentration.

Firefly luciferase activity was assessed by mixing lysates with substrate Luciferase assay reagent II (LARII) (Promega), and measuring light emission using TD 20/20 luminometer (Turner Designs, Sunnyvale, CA). Data was presented as RLU/µg of protein. β-galactosidase assay was performed from the cells extracts of samples used for both luciferase and TK activity, and were used for normalizing to β-Gal activity.

Fluorescence polarization measurements were made using a Synergy™ 4 Hybrid Microplate Reader from BioTek Instruments (Winooski, VT). Measurements were made from the top using the tungsten light source. Both parallel and perpendicular fluorescence were measured using the same 540/35-excitation and 600/40-emission filters along with a 570 nm cut off dichroic mirror. Polarization values were calculated automatically using Gen5™ Data Analysis Software (BioTek Instruments).

### Isolation of MSCs from Tri-Fusion Transgenic Mice

To obtain MSCs, 6-8 week-old tri-fusion transgenic mice were sacrificed, bone marrow was flushed from the femur, and the bone marrow derived mononuclear cells were purified by ficoll gradient. Adherent bone marrow mononuclear cells were grown in α-MEM containing 10% MSC qualified Fetal Bovine Serum (FBS) and 5µg/mL gentamycin to maintain the cells in pluripotent and undifferentiated state. The cells were used for *in vitro* and *in vivo* studies as low passage cultures (passages 4-6).

### Flow Cytometry Analysis

MSCs were incubated in 2% FBS/phosphate-buffered saline (PBS) at room temperature for 1 hour with 1 µL/mL of rat polyclonal antibody specific for mouse cluster of differentiation CD11b, CD34, CD45, CD73, CD90 and CD105 (Biolegend, San Diego, CA), subsequently stained with fluorescein-conjugated goat anti-rat IgG and IgM antibody (Invitrogen, Carlsbad, CA). Control cells were stained only by secondary antibody alone.

### Multi-lineage Differentiation of MSCs

The MSCs isolated from the Tri-Modality Reporter Mouse were used for *in vitro* and *in vivo* studies at low passage cultures (passages 4-6). To examine the differentiation potential of MSCs, cells were induced to adipocytes, chondrocytes, and osteocytes. For adipogenesis, MSCs were seeded into a 12-well plate at 10,000 cells/cm^2^ in MSC growth medium, and medium was replaced with pre-warmed adipogenesis differentiation medium (Gibco, A10070) after 24 hours after initial plating. Cells were then maintained for 10 days with media change once in every 3-4 days. The adipogenic differentiation was confirmed by Sudan III staining of lipid droplets. Similarly, for osteogenesis, MSCs were seeded in 12-well plate at 5,000/cm^2^ in MSC Growth Medium, and medium was replaced with pre-warmed osteogenesis differentiation medium (Gibco, A10072) 24 hours after plating. Cells were then maintained for 28 days with media change once in every 3-4 days. The cells were stained with alizarin red S for calcium deposition. For chondrogenesis, micromass cultures were generated by seeding 5-µL droplets of cell solution into a 12-well plate at 1.6 x 10^7^ cells/ml medium. After cultivating micromass cultures for 2 hours under high humidity conditions, warmed chondrogenesis media was added to culture vessels and incubated for 21 days with a media change every 3-4 days. The cells were stained by 1% Alcian Blue solution for the synthesis of proteoglycans by chondrocytes.

### Reporter Gene Expression in MSCs

Expression of tdTomato was observed under a Zeiss Axiovert 200M fluorescence microscope (Carl Zeiss Microimaging Inc., Thornwood, NY) with DsRed filter setting (λex, 546 nm; λem, 605 nm) and analyzed with MetaMorph software (University Imaging Corp., Downingtown, PA). For quantification of the expression level of luciferase gene, cells were seeded in black-wall clear-bottom 96-well plates was imaged using a charged coupled device camera (IVIS50, Xenogen, Alameda, CA) after the addition of 100 µL/well D-Luciferin (Xenogen, Alameda, CA, 300 µg/mL), and the peak signal (photons/second/square centimeter/steridian or p/s/cm^2^ /sr) was measured. Regions of interest (ROIs) were drawn over the cell area and quantified by using Living Image Software. To measure the truncated-TK enzyme activity, cells plated in 12-well plates were incubated with [8-^3^H]-Penciclovir, a substrate for TK, at 37° C for 1 h. The cells were harvested, and measured with a Beckman LS-9000 liquid scintillation counter as described previously [25].

### Multimodality *in Vivo* Imaging of Transplanted Cells

Nude mice were anesthetized, subcutaneously implanted with various numbers of MSCs, and then were placed in a light tight chamber equipped with a halogen light source, and whole body image was acquired for 10 s using the Xenogen IVIS optical imaging system with an excitation filter at 500–550 nm and an emission filter at 575–650 nm. For BLI, each mouse received a injection of D-Luciferin (3 mg in 100 µl PBS) and was imaged for bioluminescence as described previously. For quantification, ROIs were manually selected based on the signal intensity. The area of the ROI was kept constant and the intensity was recorded as total flux (photons per second). The same mice were then injected with ^18^F-FHBG (7.4 MBq in 200µL saline), and scanned using a microPET scanner after 1 hr. Volumetric ROIs were drawn over the interested site and the mean activities were recorded from the entire ROI. The percentage-injected dose per gram (% ID/g) tissue was calculated by dividing the ROI counts by the injected dose (decay corrected).

### Myocardial Infarction Model in Non-transgenic Littermate

Female littermates (8-weeks-old) of Tri-Modality Reporter Mouse were intubated and placed under general anesthesia with isoflurane (2%/L oxygen). Myocardial infarction was created by ligation of the mid-left anterior descending artery through a left anterolateral thoracotomy. One million MSCs were injected intramyocardially into the site near the peri-infarct zone in 20 µL of total volume (with 50% Matrigel in HBSS) 10 min after ligation (n=5). For sham-operated animals, open thoracotomy was performed, followed by injection of 1 million MSCs, but without ligation of the LAD artery (n=5). Animal protocols were approved by the Stanford University Animal Care and Use Committee guidelines. To assess longitudinal cell survival, animals were imaged for 4 weeks. BLI was performed as described previously. MicroCT imaging was initiated 1 h after injection of the radiotracer ^18^F-FHBG (7.4 MBq in 200 µL saline) followed by microPET imaging with Inveon PET/CT (Inveon, Siemens, and Munich, Germany). The microPET/CT images were reconstructed and analyzed using Inveon Research Workplace software.

### Hindlimb Ischemia Model in Nude Mice

To assess the survival in a murine model of hindlimb ischemia (HLI), the femoral artery was ligated in 10-week-old female nude mice. After ligation, 20 mice were randomized into two groups, therapeutic group receiving 1 million MSCs in 50µL PBS by intramuscular (IM) injection, control group receiving only PBS. Animal studies were approved by the Administrative Panel on Laboratory Animal Care in Stanford University. To assess longitudinal cell survival, animals were imaged for 4 weeks. BLI was performed as described previously. MicroPET/CT imaging was performed with Inveon PET/CT instrument.

### Laser Doppler Imaging

To assess the therapeutic potential of MSCs, a laser Doppler perfusion imager (Moor Instruments, Devon, United Kingdom) was used for serial noninvasive physiological evaluation of neovascularization. Mice were monitored by serial scanning of surface blood flow of hindlimbs on day 0, 7, 14, 21 and 28 after treatment. Regions of interest of the ischemic or nonischemic hindlimb were drawn in a standard fashion. The level of perfusion in the ischemic and normal hindlimbs was quantified using the mean pixel value within the region of interest, and the relative changes in hindlimb blood flow were expressed as the ratio of the left (ischemic) over right (normal) laser Doppler blood perfusion (LDBP).

### Histological Examination

Mice tissue was embedded into OCT solution (Miles Scientific), and frozen sections (10 µm) were checked under fluorescence microscope directly. To detect the fate of injected cells with tdTomato reporter gene, the tissues were immunostained with rabbit anti-RFP antibody (Clontech), mouse anti-human CD31 antibody (BD Pharmingen), and DAPI (4, 6-diamidino-2-phenylindole) nuclear counterstain. Mice tissue was also paraformaldehyde fixed, paraffin embedded, and sectioned for routine hematoxylin and eosin staining and assessed by a pathologist. At least 4 tissue sections were assessed for each animal, and 5 high-powered images were photographed for each tissue section with confocal Leica SP2 microscope (Leica Microsystems Inc., Il, USA).

### Statistical Analysis

All data are reported as mean ± standard deviation. Statistical analyses between multiple groups at each time point were performed by analysis of variance (ANOVA) with the Holm adjustment. Repeated measurements of samples over time were analyzed by repeated measures of ANOVA with the Holm adjustment. Statistical significance was accepted at P<0.05.

## Results

### Development of a ‘universal reporter’ transgenic mouse model constitutively expressing the tri-fusion reporter gene from a chicken β-actin promoter

**Figure 1:**
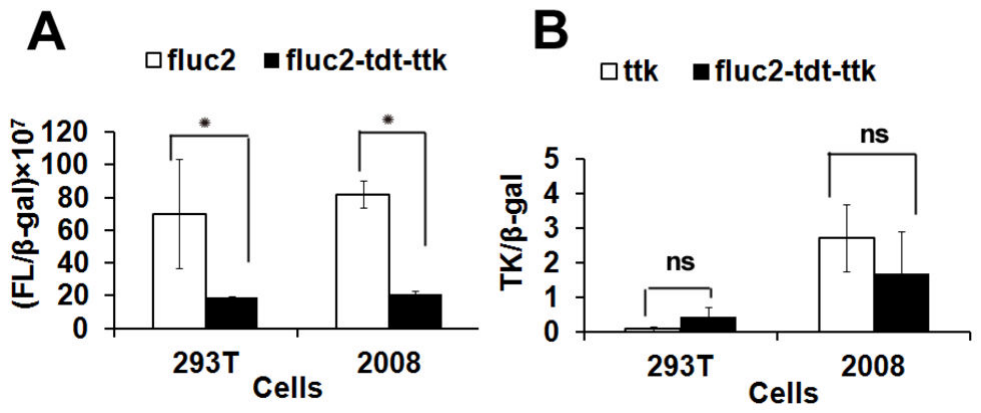
Comparative expression analysis of pcAGGS-fl2-tdt-ttk vector in 293T and 2008 cells by transient transfection. (A) A ratiometric analysis of firefly luciferase and β-galactosidase activity show 2-3 fold lower luciferase activity of the pcAGGS-fl2-tdt-ttk construct in comparison to pCMV-fl2 vector in both 293T and 2008 cells. (B) Ratiometric analysis of thymidine kinase activity and β-galactosidase activity revealed slightly higher TK in 293T cells, but slightly lower TK activity in 2008 cells by the triple fusion vector in comparison to CMV-ttk vector. (ns. -statistically non-significant and *indicates p<0.05).

We constructed a unique vector system composed of three reporter genes fusion under a chicken β-actin promoter, and used for creating a transgenic mouse model. The vector was verified for the expression of all three reporters by transient transfection studies in three different cell lines. Though luciferase activity decreased in the fusion form (as reported before [16]) (Figure 1A), the TK activity did not show much variation compare to its respective independent form (Figure 1B). Next, we created a transgenic mouse (FVB strain) that ubiquitously express fluc2-tdTomato-ttk tri-fusion reporter gene in all tissues through the Stanford Transgenic facility. After screening 52 pups by BLI, we obtained 7 positive mice (5 female mice, 2 male mice) for bioluminescence signals, and these transgenic founder mice were used for other colony expansion by using FVB male mouse. The results showed that the signals from strongly positive mice persist even after 10 generations, and no developmental abnormalities or toxicity is observed.

### Non-Invasive Imaging of the Transgenic Mouse with Bioluminescence, Fluorescence, and microPET Imaging Modalities

**Figure 2:**
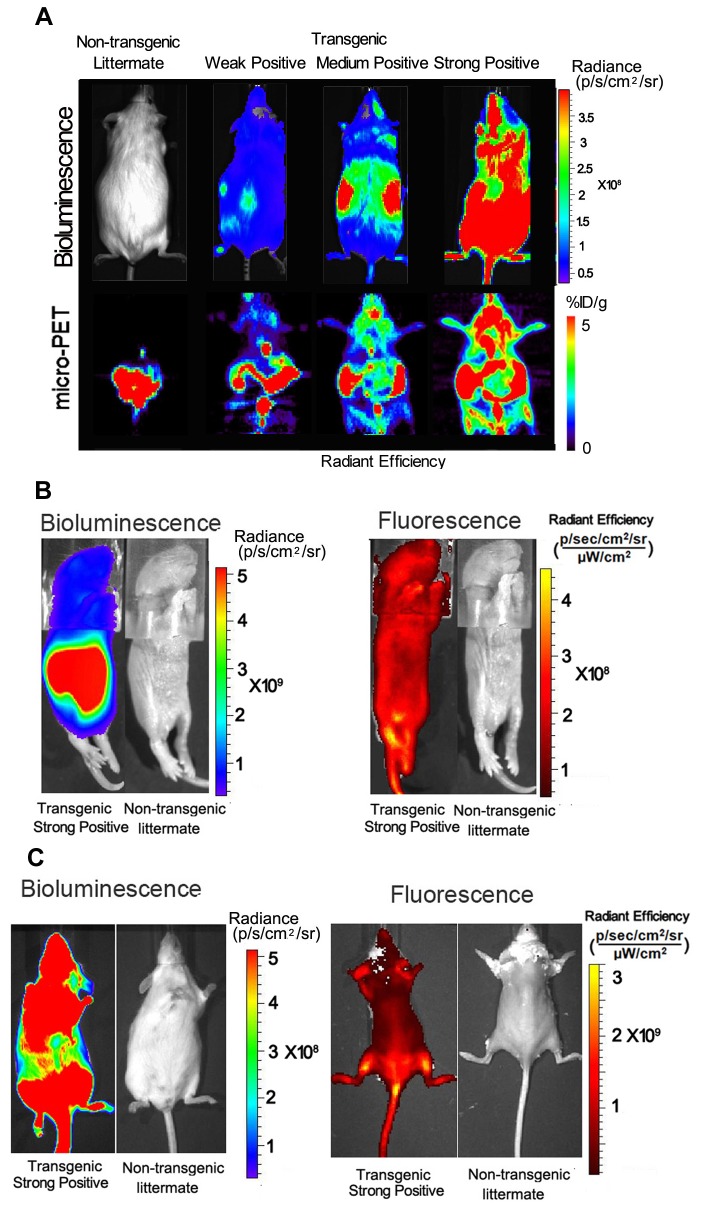
Characterization of tri-fusion transgenic mouse. (A) *In vivo* bioluminescence, micro-PET and fluorescence imaging of adult tri-fusion transgenic mice and non-transgenic littermate. Mice (N=15) with different expression levels of the tri-fusion reporter were scanned, and multimodality imaging results show that the expression level of all three genes are correlated with each other. No bioluminescence signal was found in non-transgenic littermate. Background micro-PET signal was found in heart and gastrointestinal system of non-transgenic littermate, and autofluorescence was also found in non-transgenic littermate. Transgenic positive mice show different signal levels in multimodality imaging due to the different gene expression levels. (B–C) *In vivo* bioluminescence and fluorescence imaging of strongly positive newborn and adult tri-fusion transgenic mice and non-transgenic littermates. Strong bioluminescence and fluorescence signals were found in tri-fusion transgenic mice, but not in non-transgenic littermates. p/s/cm^2^ /sr=photons per second per cm^2^ per steradian; % ID/g = percentage of injection dose per gram.

This unique gene fusion is a combination of bioluminescent, fluorescent and PET reporter genes, and thus can be imaged with all three modalities. Fifteen adult mice (12 transgenic mice express different levels of tri-fusion reporter gene and 3 non-transgenic littermates) were imaged by both bioluminescence and micro-PET imaging (Figure 2A). The results found no bioluminescence background from non-transgenic littermates, and strongly positive transgenic mice show bioluminescence signal which was beyond the saturation limit of the camera. In the subsequent micro-PET imaging, modest level of background activities was seen in the gastrointestinal regions of non-transgenic littermates. But in the case of transgenic mice, signals were broadly seen throughout the body. The imaging results show significant correlation between the expression levels of luc2 and ttk genes. There is still weak autofluorescence seen in non-transgenic adult littermate, even after we subtracted the background. We used transgenic newborn positive pups and non-transgenic littermates for bioluminescence and fluorescence imaging by IVIS200 system. Both luciferase signal and tdTomato fluorescence signal were broadly expressed throughout the body (Figure 2B). Because the autofluorescence from hairs imposes serious limitations for *in vivo* fluorescence imaging with adult mice, in subsequent experiments we removed the hairs of adult mice before imaging. In the strongly positive adult transgenic mice, both bioluminescence and tdTomato fluorescence signal were expressed broadly throughout the body (Figure 2C).

### Reporter gene expression and biodistribution in the Tri-fusion transgenic mice

**Figure 3:**
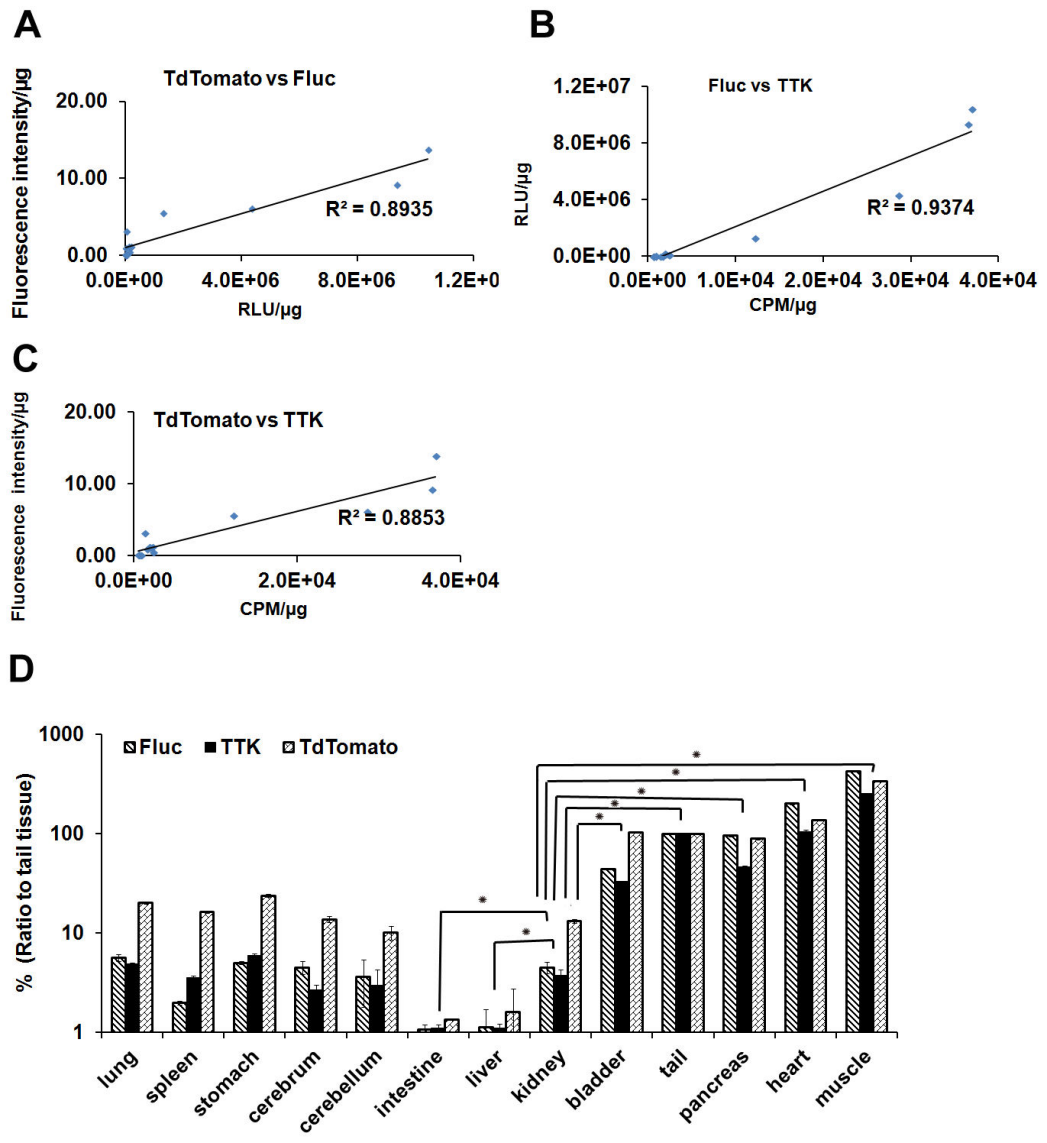
Reporter gene expression levels in transgenic mice and non-transgenic littermate. (A–C) *In vitro* luciferase assay, fluorescent assay and TK assay were performed with cell lysates from the tail of transgenic mice. Correlation of Fluc, tdTomato and TTK gene expression levels in transgenic mice and non-transgenic littermate (n=15). (D) Reporter gene expression levels in various organs from transgenic mice. Transgenic mice with high expression level of reporter gene were harvested and tested using *in vitro* assays. Muscle, heart, tail, pancreas and bladder have statistically higher expression (P<0.05) of reporter gene than kidney, while the liver and intestine have lower expression (P<0.05) (*indicates p<0.05). RLU = relative light units; cpm=counts per minute; FI = Fluorescence intensity.

After *in vivo* imaging, an *in vitro* luciferase, fluorescence, and TK activities were measured in the lysates of tissues from the tails of transgenic mice (Table 1). The results from luciferase assay revealed that the strongly positive group (mouse 12-15) showed the highest luciferase signals which is 35,000-280,000 fold higher than non-transgenics, and even the weakly positive mice (mouse 5-7) also showed 450-1100 fold higher signals than non-transgenic mice (mouse 1-4) ) (Table 1). We also found similar trend with the TK activities (strongly positive mice: 12,176.33 ± 780.64 - 36,938 ± 1,204.8 cpm/µg) which were around 20-50 fold higher than the non-transgenic mice (606.67 ± 7.2 - 610.33 ± 25.5 cpm/µg). The fluorescence signals in weakly positive mice (0.15 ± 0.05 - 3.21 ± 0.46FI/µg) is close to the non-transgenic littermates, while there were significant differences between the medium positive (0.53±0.11 - 1.23±0.65FI/µg) and strongly positive mice (5.57±1.2 - 19.25±5FI/µg). The correlation between the signal levels of FLUC2 and TTK are much higher (Figure 3B, R2 = 0.9374) compare to respective tdTomato fluorescence signal (Figure 3C, 3A, R2 = 0.8852, 0.8917). Various organs from strongly positive transgenic mice were harvested and tested using *in vitro* assays for all three reporters activities (Figure 3D). Muscle, heart, tail, pancreas, and bladder have statistically higher expression (P<0.05) of reporter genes than kidney, while the liver and intestine showed lower expression (P<0.05).

**Table 1:** Reporter gene expression levels in transgenic **mice**.

**Groups**	**mice**	**Fluc (RLU/µg)**	**TTK (cpm/ug)**	**TdTomato (FI/µg)**
**Non-transgenic**	**1**	**38.33±2**	**674.22±24.26**	**0.13±0.04**
	**2**	**37.33±1.5**	**606.67±7.2**	**0.21±0.06**
	**3**	**37.67±1.5**	**610.33±25.5**	**0.12±0.02**
	**4**	**42.33±2**	**609.67±10.4**	**0.11±0.05**
**Weak positive**	**5**	**16736.67±190.29**	**1609.33±38.8**	**0.96±0.21**
	**6**	**27251.67±1514.65**	**1390.67±86.63**	**3.21±0.46**
	**7**	**42522.5±647.19**	**910.67±38.79**	**0.15±0.05**
**Medium positive**	**8**	**127132.33±1681.79**	**2325.67±84.67**	**1.23±0.65**
	**9**	**131780.83±1080.65**	**2397.33±77.2**	**0.53±0.11**
	**10**	**158479.5±878.45**	**2040.42±118.22**	**1.12±0.14**
	**11**	**194824.3±2.08**	**1956.33±31**	**1.22±0.11**
**Strong Positive**	**12**	**1301684.33±28719.51**	**12176.33±780.64**	**5.57±1.2**
	**13**	**4356490.5±3406.78**	**28575.13±60.5**	**6.14±1.43**
	**14**	**9352680.67±25583.16**	**36462±2260.8**	**19.25±5**
	**15**	**10424357.5±4320.03**	**36938±1204.8**	**13.84±1.1**

### Reporter genes activities in MSCs isolated from tri-fusion transgenic mouse

**Figure 4:**
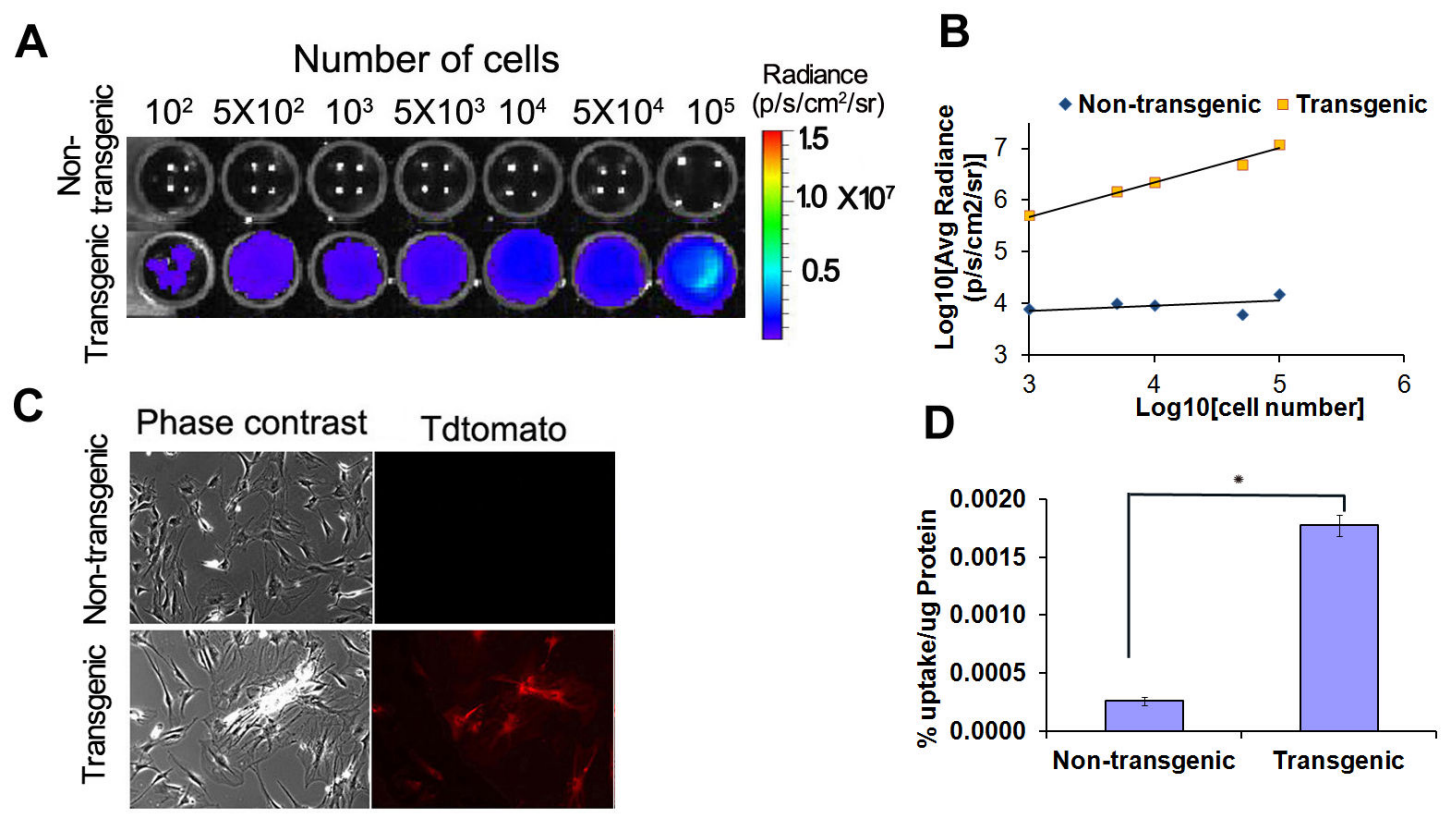
Reporter gene expression levels in MSCs isolated from tri-fusion transgenic mouse. (A) BLI of varying numbers of passage 4 MSCs (total cell count given above corresponding well, with color scale bar representing range of signal in photon per second per cm^2^ per steradian). (B) Correlation of cell numbers (x-axis) with BLI signal demonstrated linear relationships, with R^2^ values of 0.99. (C) Fluorescence imaging of passage 4 MSCs. (D) The tTK activity of passage 4 MSCs. Tri-fusion MSCs showed a significant higher cellular uptake compared with MSCs isolated from non-transgenic littermate (P<0.05). The tTK activity is expressed as (% percentage of uptake of [8-3H] penciclovir/µg protein (TRI = Tri-fusion). (*indicates p<0.05).

MSCs were harvested from 6–8 weeks old tri-fusion transgenic mice, then assessed for reporter genes activities after 4th passage. The cells plated in various numbers in a 96 well black-wall clear-bottom plate was imaged for bioluminescence signal 16 hours after initial plating by adding D-Luciferin. A relatively high bioluminescence signals were observed in cells, and the signals from as few as 100 cells can be detectable (Figure 4A) by BLI. Results showed a linear correlation between the cell number and the BLI signal (R^2^=0.9786; Figure 4B). There was no background bioluminescence signal in MSCs isolated from the non-transgenic littermate. The expression of tdTomato in P4 MSCs was observed by fluorescence microscope, and all MSCs showed tdTomato fluorescence signal (Figure 4C). The TK-activity from P4 MSCs was assayed by ^3^H-Penciclovir uptake study. Tri-fusion MSCs showed a 6.8-fold higher cellular uptake (0.177 CPM/µg protein) compared with MSCs isolated from non-transgenic littermate (0.026 CPM/µg protein; Figure 4D).

### Characterization of MSCs

**Figure 5:**
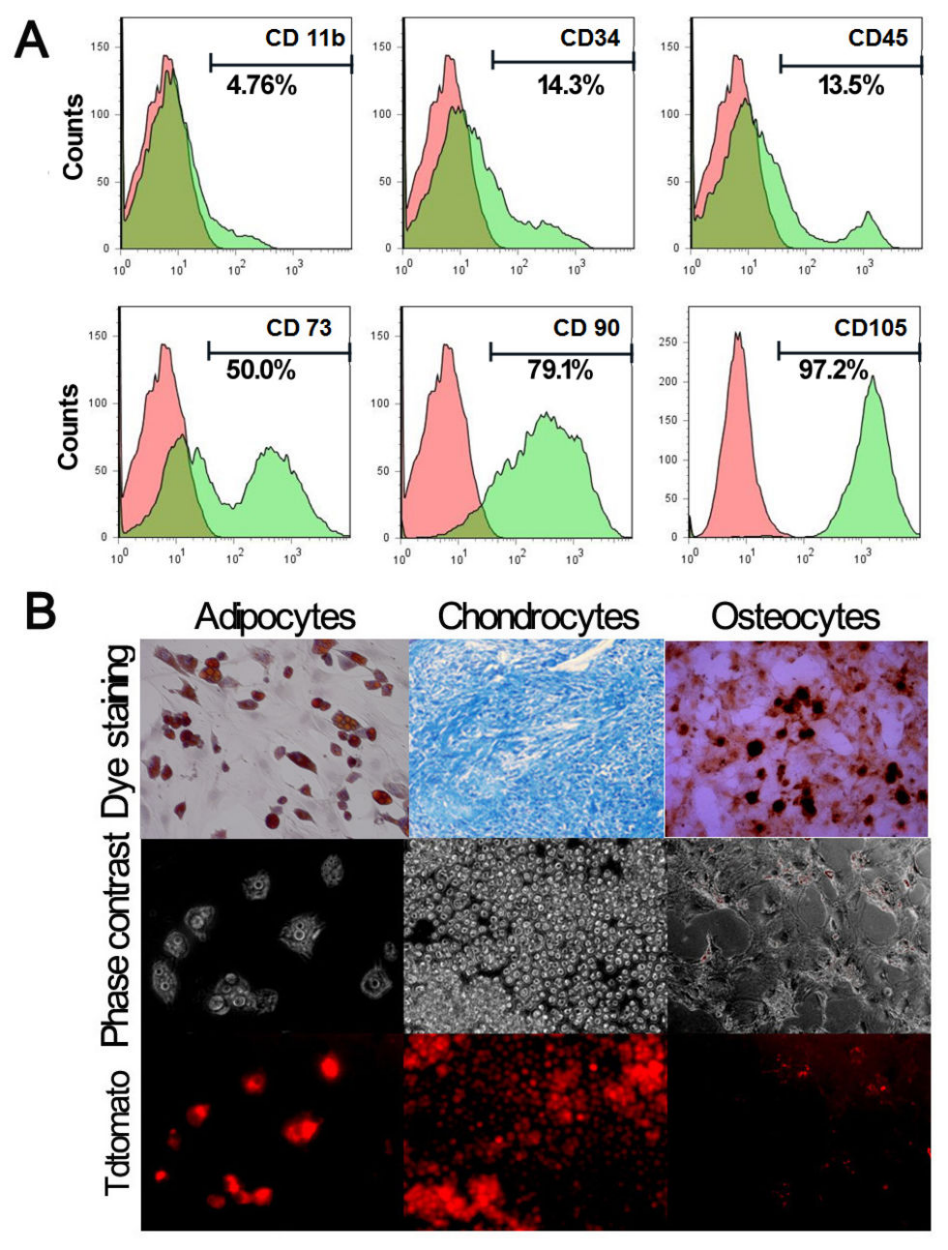
Characterization of MSCs. (A) FACS analysis of the immunophenotype profile of MSCs. MSCs are uniformly positive for extracellular matrix protein CD90, endothelial cells antigen CD105, and low-level hematopoietic progenitor cell marker CD34 and leukocyte common antigen, but are negative for macrophages monocyte antigen CD11b. (B) MSCs can differentiate into adipocytes, chondrocytes and osteocytes *in vitro* as assessed by oil Sudan III, alcian blue or alizarin S staining. Both adipocytes and chondrocytes showed fluorescent signal after differentiation.

MSCs are distinguished from hematopoietic stem cells and leukocytes by their cell surface markers. Based on the results from FACS analysis, MSCs are uniformly positive for extracellular matrix protein CD90 (79.1%), endothelial cells antigen CD105 (97.2%), and low-level haematopoietic progenitor cell marker CD34 (14.3%) and leukocyte common antigen, but are negative for macrophages monocyte antigen CD11b (4.76%) (Figure 5A). The expression pattern of cell surface markers of Tri-Modality Reporter mouse derived MSCs were consistent with other related reports in the literature [21].

To examine the differentiation potential of MSCs, cells were induced to adipocytes, chondrocytes and osteocytes *in vitro* and were assayed using Sudan III, Alcian blue and alizarin S staining, respectively. P4 MSCs were induced under adipogenic conditions for 10 days, with most of the cells producing lipid-contain adipocytes as demonstrated by oil red O staining. MSCs induced with osteogenesis medium display stereotypic increase in alizarin red S staining at Day 21. MSCs induced with chondrogenesis medium produced proteoglycans-containing chondrocytes as demonstrated by Alcian blue staining at Day 28. Both adipocytes and chondrocytes showed fluorescent signal after differentiation, while osteocytes only showed a faint fluorescence signal (Figure 5B). These results suggest that tri-fusion reporter gene expression level is different in the differentiated cells.

### *In vivo* Bioluminescence and micro-PET imaging of subcutaneously implanted MSCs in nude mice

**Figure 6:**
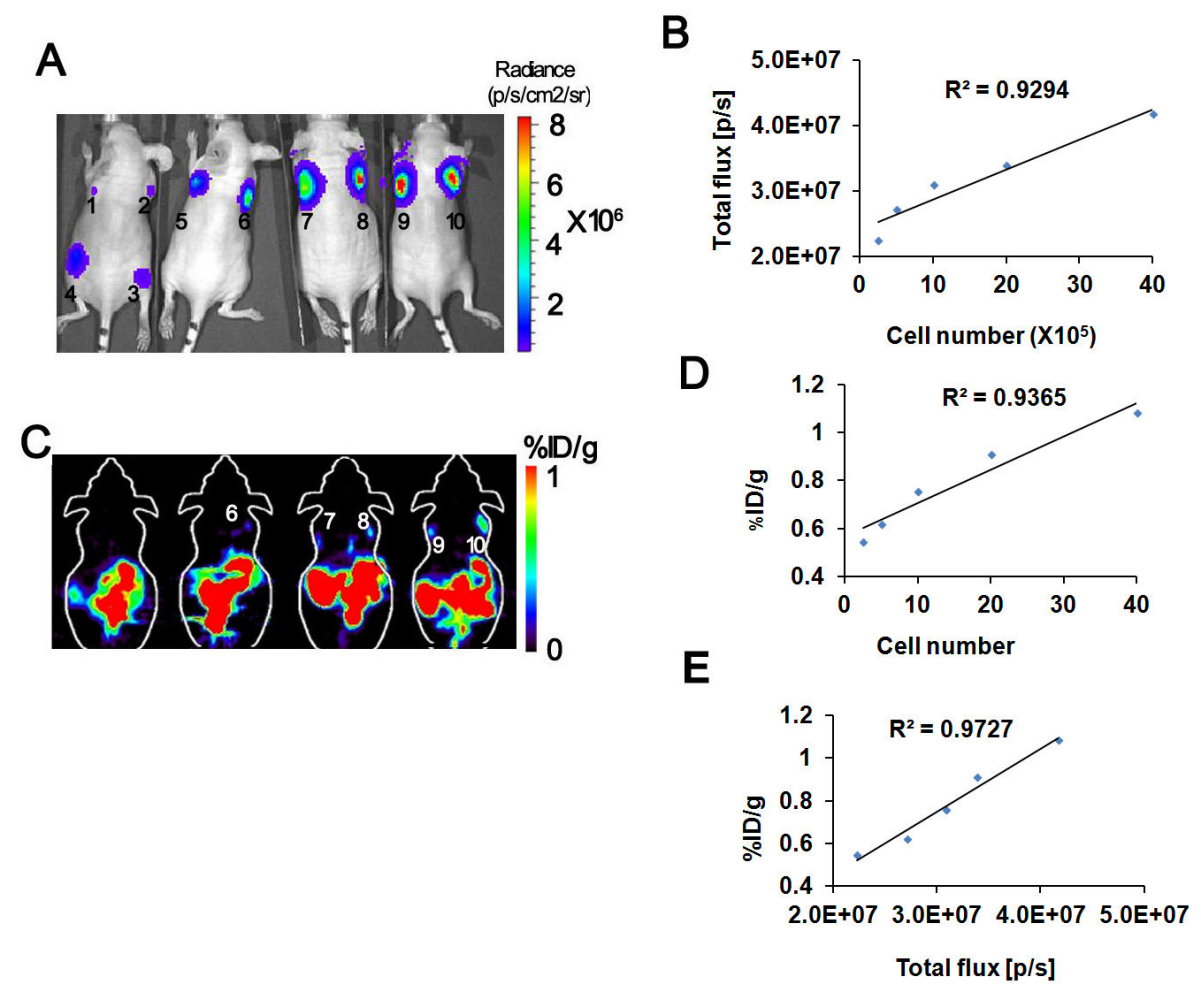
Multimodality imaging of P4 MSCs in nude mice. (A) Bioluminescence imaging of nude mice injected with P4 MSCs cells isolated from the tri-fusion transgenic mice at day 0. 1x10^3^ (A.1), 5x10^3^ (A.2), 1x10^4^ (A.3), 5x10^4^ (A.4), 1x10^5^(A.5), 2.5x10^5^ (A.6), 5x10^5^ (A.7), 1x10^6^ (A.8), 2x10^6^ (A.9), and 4x10^6^ (A.10). MSCs were injected into nude mice, and then fluorescence imaging was performed immediately. One hour later, the same mice were imaged for bioluminescence signal following intraperitoneal injection of D-Luciferin. 1X10^3^ (A.1) cells are detectable by bioluminescence imaging. (B) Correlation of cell numbers (x-axis) with bioluminescence signal. Results demonstrated linear relationship between bioluminescence signal and cell number, with R^2^ values of 0.93. (C) Micro-PET imaging of the same nude mice. Following a bioluminescence scan, the mouse was imaged in microPET using FHBG. The PET signal was found in the mouse injected with 2.5x10^5^ (C.6) or more MSCs. (D) Correlation of cell numbers (x-axis) with micro-PET signal. Results demonstrated linear relationship, with R^2^ values of 0.94. (E) Correlation of fluc and ttk gene expression. Results demonstrated linear relationship, with R^2^ values of 0.97. p/s = photons per second; % ID/g = percentage of injection dose per gram.

Our key application of creating a tri-fusion transgenic mouse was to test whether multimodality imaging can be used to track transplanted cells or organs in small living animals. We therefore injected various numbers of MSCs at different sites on the dorsal sides of 8-week-old nude mice [1x10^3^ (A.1), 5x10^3^ (A.2), 1x10^4^ (A.3), 5x10^4^ (A.4), 1x10^5^(A.5), 2.5x10^5^ (A.6), 5x10^5^ (A.7), 1x10^6^ (A.8), 2x10^6^ (A.9), and 4x10^6^ (A.10) (Figure 6)]. The mice were first scanned using the cooled CCD camera for fluorescence followed by a bioluminescence after injection of 100 µl D-Luciferin (30 mg/mL) intraperitoneally. Fluorescence imaging of these mice reveals that a minimum of 5x10^5^ MSCs can be visualized (data now shown). With BLI of the same nude mice, we found that even 1x10^3^ MSCs can be detectable (Figure 6A). We found significant correlation (r^2^=0.9294) between the injected cell numbers and the bioluminescence signal (Figure 6B). Two hours after BLI, ^18^F-FHBG (7.4 MBq in 200 µL saline) was administered via the tail vein. Micro-PET scanning was performed one hour after the injection, and we found that a minimum of 2.5x10^5^ (site 6) MSCs can be visualized by PET imaging (Figure 6C). There is also a linear correlation between the PET signal with the cell numbers (R^2^= 0.9365; Figure 6D). The high correlation of fluc and ttk gene expression in the *in vitro* assay predicted the high correlation of bioluminescence signal and micro-PET signal for *in vivo* imaging (R^2^ =0.9727; Figure 6E).

### Bioluminescence and microPET imaging of MSCs survival in mice with acute myocardial Infarction

**Figure 7:**
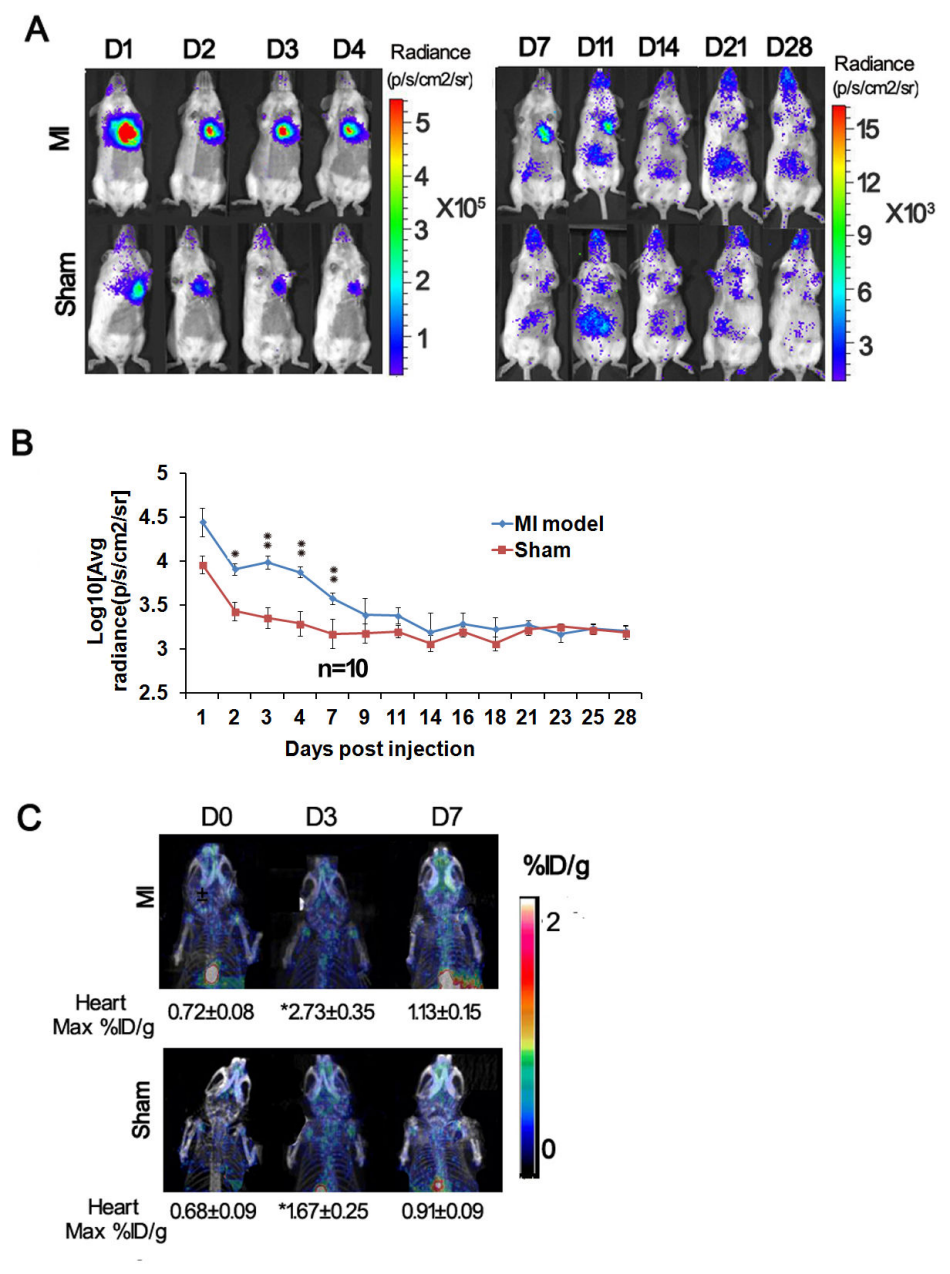
Bioluminescence imaging of transplanted MSCs in mice with myocardial infarction (MI) and sham model. (A–B) Mice in both sham and MI groups received 1 million MSCs by intramyocardial injection (n=5). To assess longitudinal cell survival, animals were imaged for 4 weeks. A representative image of mice injected with MSCs showed significantly higher bioluminescence signal in MI heart (top) than in sham heart (bottom) signals at day 2 (p<0.05), day 3, day 4, day 7 (p<0.01). In both groups, bioluminescence signals decreased to background levels after 4 weeks. p/s/cm^2^ /sr=photons per second per cm^2^ per steradian; p/s = photons per second. (C) Micro-PET imaging of the same mice. Following a bioluminescence scan, the mouse was imaged in microPET using FHBG on day 0, 3 and 7. Higher microPET signal was found in the infarcted heart in day3. (٭٭P<0.01, * P<0.05).

To monitor MSCs survival in a specific disease model, non-transgenic littermate mice with acute myocardial infarction induced by coronary ligation subsequently underwent intramyocardial injection of 1 million Tri-Modality Reporter mouse-derived P4 MSCs. A sham model was made only by intramyocardial injection of 1 million MSCs into non-transgenic littermate without infarction. To assess longitudinal cell survival, animals were imaged for 4 weeks. A representative image of mice injected with MSCs showed higher bioluminescence signal in MI heart (top) than in sham heart (bottom) (Figure 7A). Quantification of cell signal in the heart showed significantly higher signals in MI heart at day 2 (p<0.05), day 3, day 4, and day 7 (p<0.01) (Figure 7B). Bioluminescence signals decreased gradually from week 1 to 4, which indicated MSCs can be tracked by BLI (Figure 7A). At days 0, 3 and 7, ^18^F-FHBG-micro-PET imaging was performed following bioluminescence imaging. The maximum % ID/gm tissues of ^18^F-FHBG accumulation in heart was found increased after MSCs injection on day 3, and gradually dropped to background level on day 7. In comparison, the MI heart had higher maximum % ID/gm tissue of ^18^F-FHBG accumulations on day 3 compared to sham heart (p<0.05; Figure 7C). We did not see any significant PET signal in the infarction zone following PET scan on day 10 (data not shown).

### Bioluminescence and microPET imaging of MSCs survival in the nude mice with ischemic hindlimb

**Figure 8:**
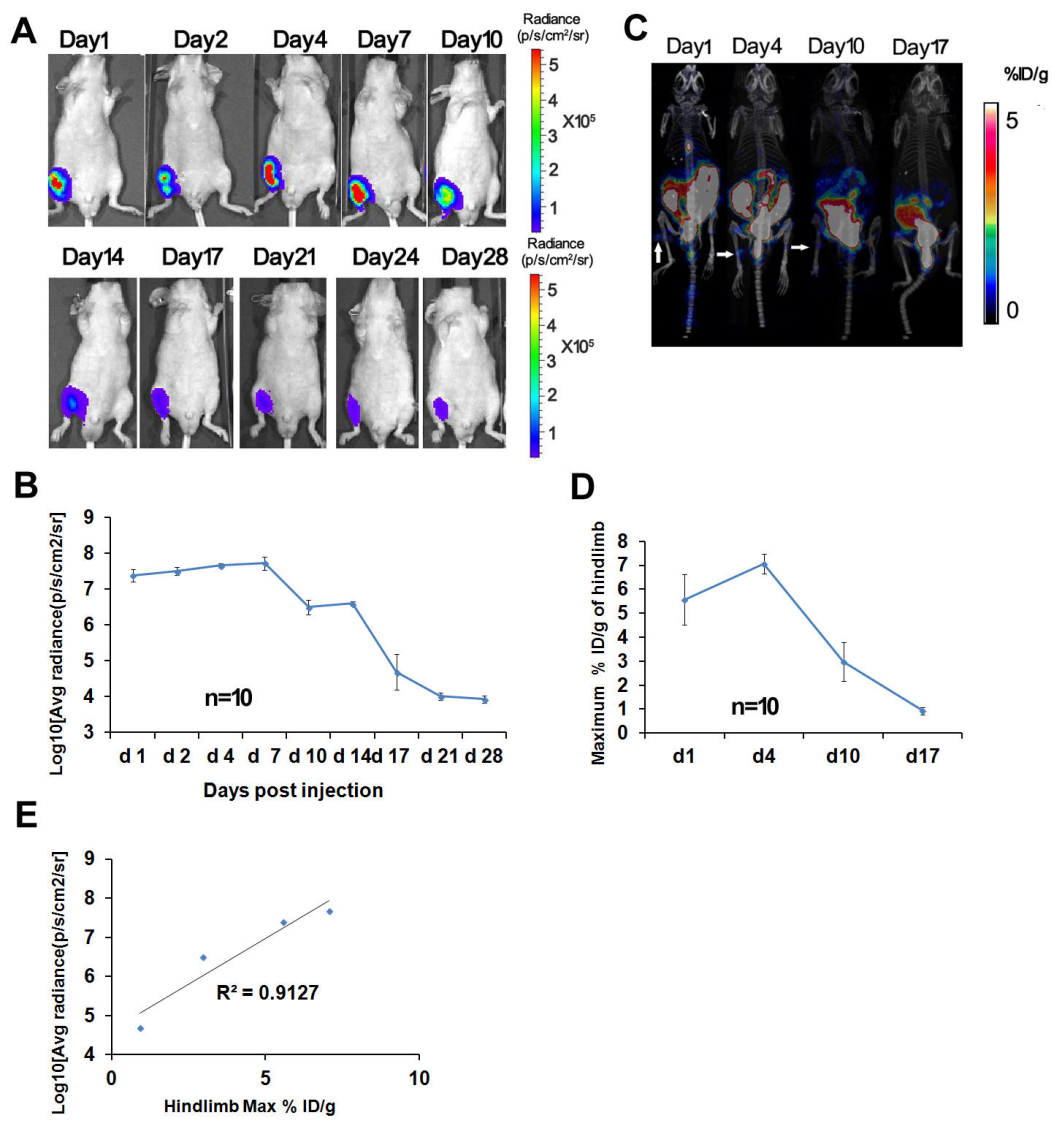
Survival of MSCs engrafted into the ischemic hindlimb. (A–B) Bioluminescence imaging of transplanted MSCs in mice with ischemic hindlimb (HLI). A representative mouse received 1 million MSCs by intramyocardial injection (n=10). To assess longitudinal cell survival, animals were imaged for 4 weeks. BLI signals were increased 5% in the first week, and then dropped to background levels after 4 weeks. p/s/cm^2^ /sr = photons per second per cm^2^ per steradian; p/s = photons per second. (C–D) Micro-PET imaging of the same mice. Following a bioluminescence scan, the mouse was imaged in microPET using FHBG on day 1, 4, 10 and 17. (E) Correlation of bioluminescence signal and FHBG accumulation in the MSCs injected hindlimb. Results demonstrated linear relationship, with R^2^ values of 0.9127. p/s = photons per second; % ID/g = percentage of injection dose per gram.

To noninvasively assess the survival of engrafted MSCs in hindlimb ischemic model, MSCs were transplanted in ischemic limb of nude mice and PBS was injected into ischemic limb of control mice. All the mice were imaged for 4 weeks. As representative mouse images shown in Figure 8A & B, the BLI signal from the implanted cells are initially increasing slowly until day 7 (5.684×10^7^ [p/s/cm^2^ /sr]), and then it started gradually dropping down by day 28 (8.685×10^3^ [p/s/cm^2^ /sr]). In contrast, there was not any background signal in the PBS injected control group (data not shown). This data confirms our above results in the mouse MI model, that BLI can be used to sensitively track Tri-Modality Reporter Mouse MSCs implanted in animals for therapeutic purpose. In addition, we performed ^18^F-FHBG-microPET imaging of the mice on days 1, 4, 10, and 17-post surgery (Figure 8C). At day 4 following MSCs implantation, there was a slight increase in the maximum % ID/g of ^18^F-FHBG accumulation in the hind limb, and it declined on day 10 and 17, consistent with the results of BLI scanning (Figure 8D). There is a linear correlation between bioluminescence signal, and ^18^F-FHBG accumulation in the MSCs implanted hindlimb (R^2^= 0.9127; Figure 8E).

### MSCs mediated re-perfusion in the ischemic hindlimb

**Figure 9:**
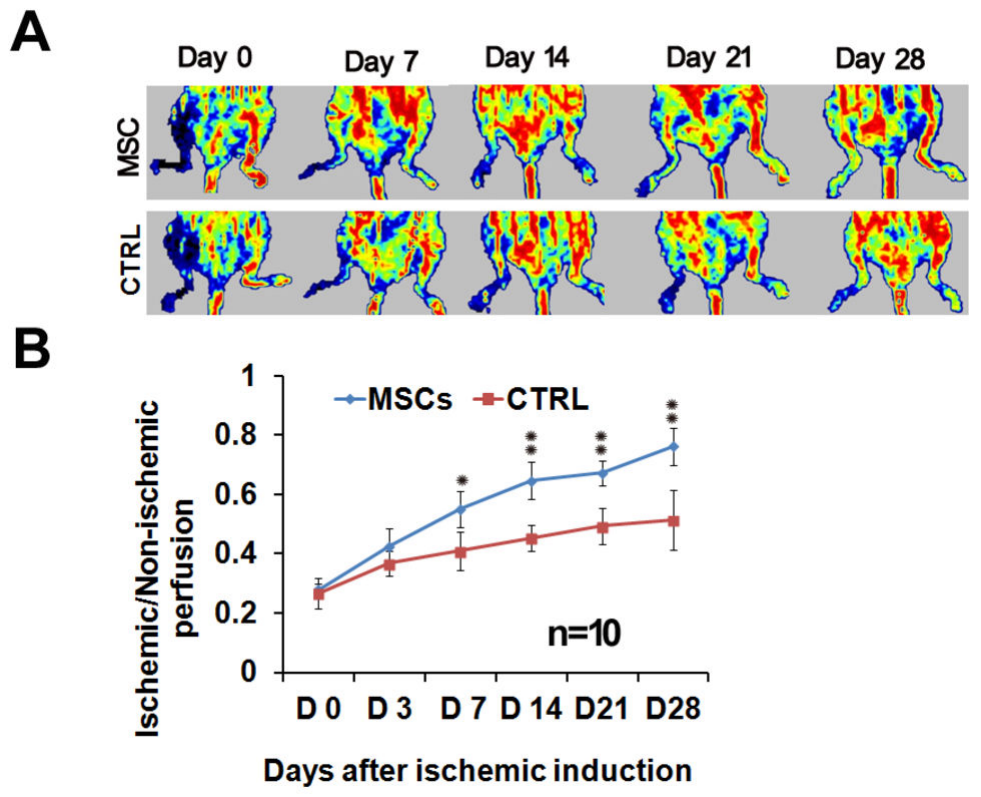
MSCs engraft into the ischemic hindlimb and restore perfusion. (A) The blood flow was assessed by laser Doppler perfusion imaging (LDPI) at days 0, 7, 14, 21 and 28 after surgery. Normal perfusion is displayed as red, while low or absent perfusion is displayed as dark blue. (B) LDPI results showed that blood flow in the ischemic hindlimb was decreased equally in both groups immediately after surgery (D0). Over the subsequent 28 days, blood perfusion of the ischemic hindlimb notably improved in the treatment group. At days 14, 21, and 28 the perfusion index is significant higher in the treatment group than control group. (٭٭P<0.01, * P<0.05).

Laser Doppler perfusion imaging (LDPI) analysis revealed that blood re-perfusion in ischemic limbs was significantly improved in the MSCs transplantation group compared with the control group (Figure 9A). One week after treatment, the relative ratios of blood flow (ischemic to normal limb) were improved to 0.551±0.062 in the MSCs transplantation group (n=10) compare to control (0.413±0.065) (n=10; P<0.05; Figure 9B). The ratio of blood re-perfusion significantly improved in two weeks after MSCs transplantation (MSC group: 0.647±0.064, Sham group: 0.454±0.043, n=10; P<0.01) and further in four weeks (MSCs group: 0.761±0.051, Sham group 0.513±0.101, n=5; P<0.01) (Figure 9B).

### Histological analyses of MSCs transplanted ischemic limbs

**Figure 10:**
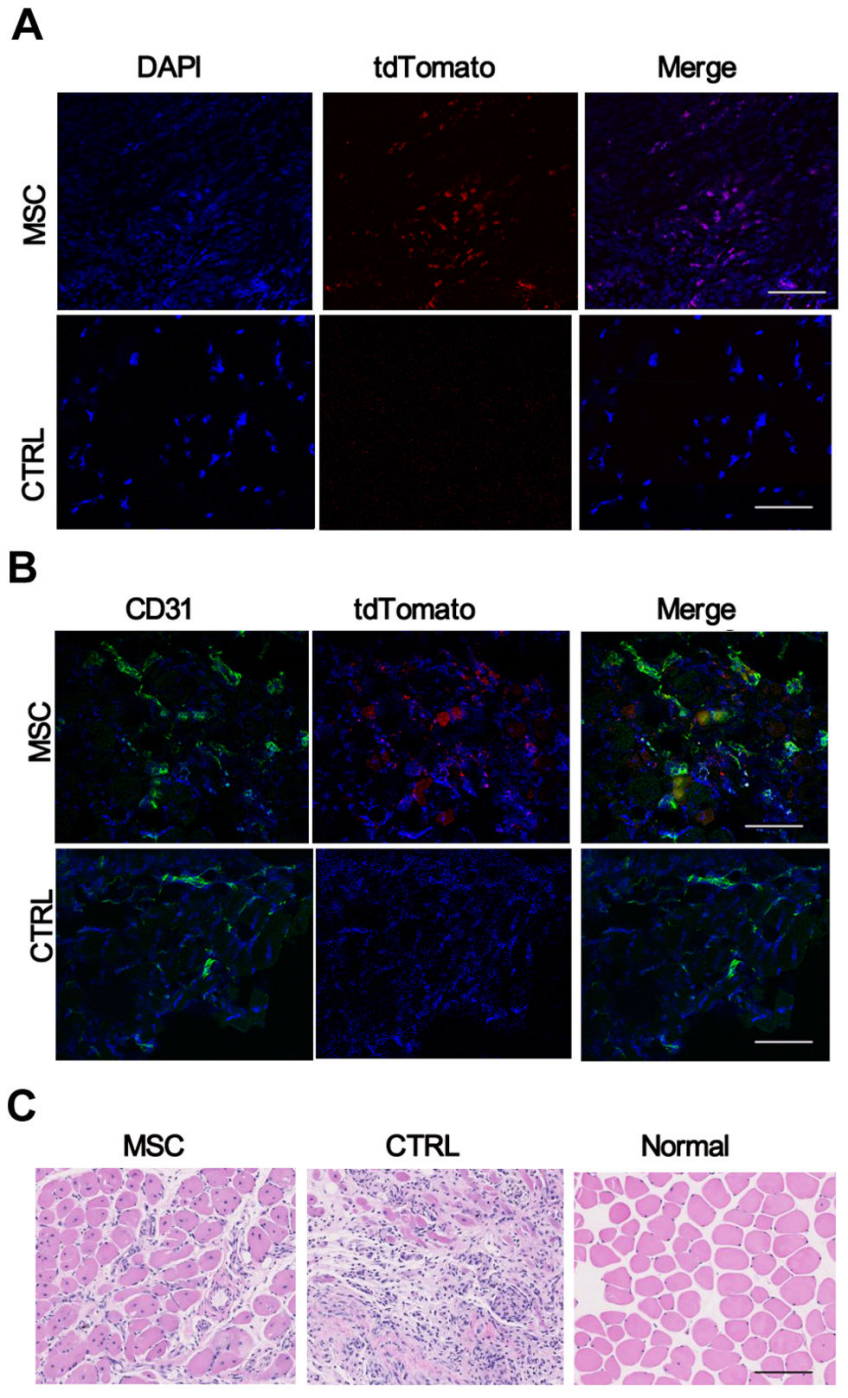
Histological analysis of MSCs survival in ischemic hindlimb. (A) Fluorescence images confirmed the residence of MSCs (red) in ischemic hindlimb in MSC group (upper panel), but not in PBS control group (lower panel). (B) Immunofluorescence staining of endothelial marker CD31 (green), tdTomato (red), and DAPI nuclear dye (blue) suggested that the surviving MSCs in the ischemic hindlimb associated with capillaries. (C) Representative histology from histological analyses of normal limbs and ischemic limbs retrieved 4 weeks after treatment. H&E staining of the peri-infarct zone demonstrate massive muscle degeneration in the ischemic regions of control limbs. In contrast, muscles in the ischemic limbs of the MSCs transplantation group were protected after cell transplantation (Scale bars = 100µm)..

Three weeks after the MSC transplantation, mice were sacrificed; ischemic hind limbs were collected and embedded in OCT. The frozen sections were sliced (1µM) and checked under fluorescence microscope directly. Cells with tdTomato signal were found in the ischemic regions, but no red fluorescent cells in the control group (Figure 10A). These cells can also be identified by double-staining for CD31 (green) and tdTomato (red), and then counterstained by DAPI as shown in Figure 10B. However, relatively few surviving MSCs were identified around the capillaries, and less frequently around the larger vessels, consistent with *in vivo* imaging data which shows weak cell signal at later time points. Ischemic hindlimbs were also collected 4 weeks after treatment, and the results of hematoxylin and eosin (H&E; original magnification 400) staining shows massive muscle degeneration in the ischemic regions of control limbs. Infiltration of numerous granulocytes and neutrophils, indicative of tissue inflammation after ischemia, was observed in the ischemic regions. In contrast, muscles in the ischemic limbs of the MSCs transplantation group display substantially reduced tissue degeneration and minimal fibrosis (Figure 10C).

## Discussion

The multimodality fusion reporter genes offers possibility to monitor molecular events ranging from a single live cell (fluorescence imaging, Fluorescence activated cell sorting (FACS)) to the multicellular environment of living mice with higher sensitivity (bioluminescence imaging), and with tomographic information from deep tissues (microPET imaging). In this study, we have generated a transgenic mouse that expresses the tri-fusion reporter gene, fluc2-tdTomato-ttk, driven by a chicken β-actin promoter. We used reporter genes with higher sensitivity to construct the system (fluc2: an improved mammalian codon optimized bioluminescent reporter; tdTomato: an improved red shifted fluorescent protein, and ttk: a truncated herpes simplex virus type 1 sr39 thymidine kinase for enhanced nuclear and cytoplasmic distribution of the protein). This system allows one to image any cell isolated from this transgenic mouse using bioluminescent, fluorescent, and PET imaging techniques. This Tri-Modality Reporter mouse can be used as a source of donor cells and organs in various critical research areas, such as tissue engineering, organ transplantation/rejection, and stem cell research to increase our understanding of regenerative mechanisms. We tested this mouse model on several applications of stem cell study, and found that MSCs isolated from transgenic mice retain their differentiation potential while expressing all three reporter genes. In both the mouse myocardial infarction model and hindlimb ischemia model, transplanted MSCs can be serially and sensitively monitored by multimodal molecular imaging techniques. Our results demonstrated that the Tri-Modality Reporter Mouse can potentially serve as a useful source for most cell population pre-labeled with a tri-fusion reporter gene.

The FVB strain was utilized to create a Tri-Modality Reporter Mouse due to the fact that white fur is better for BLI as compared to pigmented mice. Biological variability of skin pigmentation was found to dramatically affect collected bioluminescent signal emits through skin as reporter in a previous study [26]. The investigators have demonstrated that an animal with highly pigmented skin can attenuate an average of 90% signals compare mouse with no skin pigment. In addition, Chicken β-actin promoter was used to drive the tri-fusion gene in the Tri-Modality Reporter Mouse, as this promoter is able to drive constitutive expression in all mouse tissues without any gene silencing, which is normally observed with viral promoters, and is widely used in transgenic animal research [27]. Even though β-actin promoter enables the reporter gene to ubiquitously express in all tissues, we found variation in the expression levels in different tissues. We found that muscle, heart, tail, pancreas and bladder have higher expression (P<0.05) of reporter genes than other tissues, while the liver and intestine have less expression. This gene expression pattern is consistent with previous study [27].

Luciferase and ttk gene expression in MSCs can be quantitatively detected by luciferase assay and TK assay, respectively, and the expression of tdTomato can be observed under fluorescence microscope. The MSCs isolated from the Tri-Modality Reporter Mouse efficiently differentiated in to adipocytes, chondrocytes and osteocytes without any interference from the tri-fusion reporter gene, and these differentiated cells were efficiently observed under different imaging modalities (bioluminescence, fluorescence, and microPET). Fluorescent signals were found in both adipocytes, and chondrocytes, but after differentiation osteo cells had very low fluorescence signal. This result is likely due to different actin mRNA levels in different cell type. A previous study which examined the effect of osteoblast differentiation on the expression of beta-actin gene in MC3T3-E1 cells showed reduction in the expression of the β-actin gene with culture days after osteoblast differentiation [28].

The ttk gene in this Tri-Modality Reporter Mouse has a deletion of the first 45 amino acids that contains a nuclear localization signal and a cryptic testis-specific transcriptional start point [29,30]. This deletion leads to higher cytoplasmic distribution of TK enzyme, thus resulting in more TK activity due to the availability of a greater amount substrate (FHBG). This deletion mutant also overcomes the problem of male sterility in transgenic mice carrying the complete thymidine kinase gene due to production of a shorter transcript in testis from a cryptic transcriptional initiation site present in the first 135 bp of the gene [31].

Among the three modalities, BLI has the highest signal to background ratio and thereby demonstrate maximum sensitivity. Even as few as 1x10^3^ Tri-Modality Reporter mouse MSCs were detected by bioluminescence as compared with 5x10^5^ or more MSCs needed for fluorescence imaging. However, lower photon generation limits BLI at single cell level, whereas a single fluorescent cell can easily be observed under fluorescence microscope. We could detect a minimum of 2.5x10^5^ MSCs by micro-PET imaging whose sensitivity stays between that of the fluorescence and bioluminescence imaging. With high sensitivity, good spatial resolution and its tomographic nature, micro-PET holds great promise for reporter gene use in large animals including human. Our lab has done studies in normal human volunteers with [^18^F]-FHBG, a substrate for HSV1-sr39TK, and shown its safety and proven its potential for imaging reporter gene expression in humans [32]. We have also done a pilot study in T-cells tagged with a PET reporter gene and injected into glioblastoma patients [33]. Therefore, the current results support for the applicability of fluorescence component for cell imaging/sorting with limited *in vivo* imaging, bioluminescence for small animal imaging involving a very few number of cells, and PET for tomographically imaging living subjects including larger animals and humans.

MSCs have the ability to differentiate into multiple cell lineages, and can expand in large numbers, and hold the potential to repair or reconstitute a wide array of organ damage including heart. The mechanisms for homing and therapeutic effects of MSCs are still unclear, because in most of the clinical studies, the therapeutic effect was indirectly determined by functional recovery of the organs. These parameters do not provide any information on the longitudinal survival of transplanted stem cells. The success of clinical application of MSCs requires methods which can determine the biodistribution, survival, and proliferation of the transplanted MSCs in living animals. In a previous study, MSCs labeled with MRI contrast reagent-Feridex and fluorescent marker-cell tracker, and then radiolabeled with ^111^In oxine were systemically transplanted to a dog with MI heart and scanned by SPECT/CT and a MRI scanner. SPECT was able to visualize the radiolabeled cells for up to 7 days from injection, but MRI could not detect the cells [34]. The development of indirect labeling of cells using reporter genes overcomes the limitation for the loss of label through dilution during cell division that occurs with the direct labeling process. In another study, the investigators have shown that hepatocyte growth factor (HGF) and vascular endothelial growth factor (VEGF) has prolonged the transplanted MSCs survival up to 9 days compared to 7 days in the MSCs only group [35] by BLI. This technique was advanced in viral transduction methods for gene transfer and stable expression. Adipose-derived multipotent cells were transduced with lentivirus with tri-fusion reporter gene and imaged by BLI for 25 days and micro-PET for 10 days [23]. Furthermore, the development of GFP- expressing transgenic animals has resulted as a reliable source of labeled cell populations with sufficient stability of the reporter gene [27]. However, to check the fluorescence signal, cells or tissues have to be isolated from the sacrificed animals. The development of the luciferase transgenic mice makes it possible to dynamically monitor the transplanted cells by non-invasive imaging [12]. This transgenic mouse-L2G85 expresses the marker gene in all cells and tissues, and it can serve as a universal cell donor for cell transfer experiments into wild type hosts or to immunodeficient animals. Hematopoietic stem cells, bone marrow mononuclear cells and MSCs were isolated from the L2G85 transgenic mice constitutively expressing both Fluc and eGFP reporter gene. The isolated stem cells were widely used to study their rejection and survival in transplantation [13], engraftment in ischemic myocardium [36,37], and engraftment in tumor-bearing mice [21].

In the above mentioned studies, the Fluc has been used for high-throughput BLI of stem cell survival, proliferation, and migration in small animals, and the eGFP has been used for single cell fluorescence microscopy imaging, and for isolating stable clones by FACS. However, both fluorescence and BLI rely on low energy light photons which become attenuated within deep tissues and thus are not applicable for large animal and human studies. In our study, we have created a transgenic mouse that expresses the tri-fusion reporter gene driven by a chicken β-actin promoter carrying fluc2, tdTomato and ttk reporter genes. This allows us to image transplanted MSCs in nude mice with micro-PET imaging. We found that 2.5x10^5^ or more MSCs derived from Tri-Modality Reporter mouse can be visualized by PET imaging. There is a high linear correlation between the PET signal and cell number, with an R^2^ value of 0.94. MSCs were also transplanted to the non-transgenic littermate with myocardial infarction, and our results demonstrated higher micro-PET signal in MI heart (right) than in sham heart (left) on day 3. Micro-PET scanning was also performed on days 7 and 10, but we did not see any signal in the infarction area (data not shown). In another application, MSCs were transplanted directly to the ischemic hindlimb of nude mice and we found that the PET signal can last for up to two weeks after transplantation. At day 4 following MSCs injection, there was a slight increase of PET signal in the hindlimb, and then it declined at day 10 and 14, consistent with the results of BLI. Thus, MSCs isolated from this Tri-Modality Reporter Mouse can survive in both immunodeficient nude mouse and in FVB littermate and the engraftment can be tracked by multimodality imaging.

Although majority of studies involving MSC transplantation have delivered the cells directly to the tissue of interest, there is also the need for the systemic delivery of MSCs. In future, we will perform studies on the systemic delivery of various stem cells isolated from Tri-Modality Reporter mouse, as the results from this study is promising and showed as few as 1,000 cells homing in to a particular site can be sensitively imaged by bioluminescence. Neural stem cells and macrophage cells can also be isolated from Tri-Modality Reporter Mouse, and studied by imaging the higher level of reporter gene expressed by the cells. These cells will be used to address problems in other related disease model. In the future, other tissue specific promoters may be utilized for the development of tri-fusion transgenic mice to study the programmed gene expression in different cell types, tissues and organs of interest, and for developing different cell culture models or for use in gene therapy. The reporter transgenic mouse can also be used to investigate the expression of any gene of interest by constructing the tri-fusion gene to the related promoter. The PET reporter gene will make it possible to track the transplanted cells in large animals and humans and it will be likely lead to translational approaches which holds great promise for advances in a number of fields where there are clinical needs.

In conclusion, we developed a live animal platform for monitoring and validating stem cell survival and proliferation as well as tissue engineering and organ transplantation using multimodal non-invasive imaging technologies. This Tri-Modality Reporter mouse will be a useful source of donor cells and organs for various research areas such as stem cell research, tissue engineering research, and organ transplantation.
